# Close of Volume Thirteen

**Published:** 1874-07

**Authors:** 


With the present number we close Volume Thirteen. We have to express to our friends our thanks for their support in the past, and ask their continued good will in the future.

